# Platelet function, coagulation and fibrinolysis in patients with previous coronary and cerebrovascular ischemic events

**DOI:** 10.6061/clinics/2019/e1222

**Published:** 2019-09-19

**Authors:** Carlos José Dornas Gonçalves Barbosa, Renata de Souza Barreiros, André Franci, Flávia Bittar Brito Arantes, Remo Holanda de Mendonça Furtado, Célia Maria Cassaro Strunz, Tânia Rúbia Flores da Rocha, Luciano Moreira Baracioli, José Antônio Franchini Ramires, Roberto Kalil-Filho, José Carlos Nicolau

**Affiliations:** I Instituto do Coracao (InCor), Hospital das Clinicas HCFMUSP, Faculdade de Medicina, Universidade de Sao Paulo, Sao Paulo, SP, BR; II Hospital do Coracao do Brasil, Rede D'Or Sao Luiz, Brasilia, DF, BR; III Faculdade de Medicina da Universidade de Uberlandia, Uberlandia, MG, BR; IV Hospital das Clinicas HCFMUSP, Faculdade de Medicina, Universidade de Sao Paulo, Sao Paulo, SP, BR

**Keywords:** Platelet Aggregation, Blood Coagulation, Fibrinolysis, Coronary Disease, Stroke

## Abstract

**OBJECTIVES:**

Ischemic stroke (IS) or transient ischemic attack (TIA) history is present in 4-17% of patients with coronary artery disease (CAD). This subgroup of patients is at high risk for both ischemic and bleeding events. The aim of this study was to determine the role of platelet aggregability, coagulation and endogenous fibrinolysis in patients with CAD and previous IS or TIA.

**METHODS:**

A prospective case-control study that included 140 stable CAD patients divided into two groups: the CASE group (those with a previous IS/TIA, n=70) and the CONTROL group (those without a previous IS/TIA, n=70). Platelet aggregability (VerifyNow Aspirin® and VerifyNow P2Y12®), coagulation (fibrinogen and thromboelastography by Reorox®) and endogenous fibrinolysis (D dimer and plasminogen activator inhibitor-1) were evaluated.

**RESULTS:**

Patients in the CASE group presented significantly higher systolic blood pressure levels (135.84±16.09 *vs* 123.68±16.11, *p*<0.01), significantly more previous CABG (25.71% *vs* 10%, *p*=0.015) and significantly higher calcium channel blocker usage (42.86% *vs* 24.29%, *p*=0.02) than those in the control group. In the adjusted models, low triglyceride values, low hemoglobin values and higher systolic blood pressure were significantly associated with previous IS/TIA (CASE group). Most importantly, platelet aggregability, coagulation and fibrinolysis tests were not independently associated with previous cerebrovascular ischemic events (CASE group).

**CONCLUSION:**

Platelet aggregability, coagulation and endogenous fibrinolysis showed similar results among CAD patients with and without previous IS/TIA. Therefore, it remains necessary to identify other targets to explain the higher bleeding risk presented by these patients.

## INTRODUCTION

Ischemic stroke (IS) or transient ischemic attack (TIA) history is present in approximately 4-8% 1-3 of patients with acute coronary syndrome (ACS) and approximately 4-17% of patients with stable atherosclerotic disease (CAD) 4-6.

This patient population has a higher prevalence of cardiovascular risk factors and a higher prevalence of established cardiovascular disease compared to patients without previous ischemic cerebrovascular events (IS/TIA). These characteristics imply a high risk for major cardiovascular events, including cardiovascular mortality 4-7.

On the other hand, patients with previous IS/TIA also have an increased incidence of major bleeding events, especially when subjected to the more potent modern antithrombotic regimens, which include some of the new antiplatelet drugs that are contraindicated in this population 8-10.

Platelet aggregability, the coagulation system and endogenous fibrinolysis play key roles in the "ischemic-hemorrhagic" balance. However, little is known about the roles of these factors specifically in patients with prior cerebrovascular events.

The purpose of this study was to evaluate whether patients with CAD and previous IS/TIA show any differences in terms of platelet aggregation, coagulation tests or endogenous fibrinolysis in comparison with patients without these complications, which could explain, at least in part, the increased bleeding risk in these individuals.

## METHODS

Study design: This study was designed as a case-control study (with a 1:1 allocation ratio). Individuals in the case (those with a previous IS/TIA) and control groups were retrospectively selected and matched for sex, age, type of previous ACS (STEMI, NSTEMI and UA), and time between the ACS and inclusion in the study. Ethics approval was obtained from the local institutional review board.

Inclusion criteria: The inclusion criteria included a prior ACS (over 12 months prior to inclusion), a history of IS/TIA prior to the diagnosis of ACS, chronic use of aspirin since the diagnosis of ACS, and signing of the inform consent form.

Exclusion criteria: The exclusion criteria included a prior hemorrhagic stroke, current dual antiplatelet therapy or anti-inflammatory nonsteroidal therapy, any thrombophilia or coagulopathy, thrombocytopenia (<100,000/mm^3^), thrombocytosis (>450,000/mm^3^), PCI or CABG in the last 6 months, severe renal impairment (GFR <30 mL/min by MDRD) and any terminal illness.

**Figure 1:**
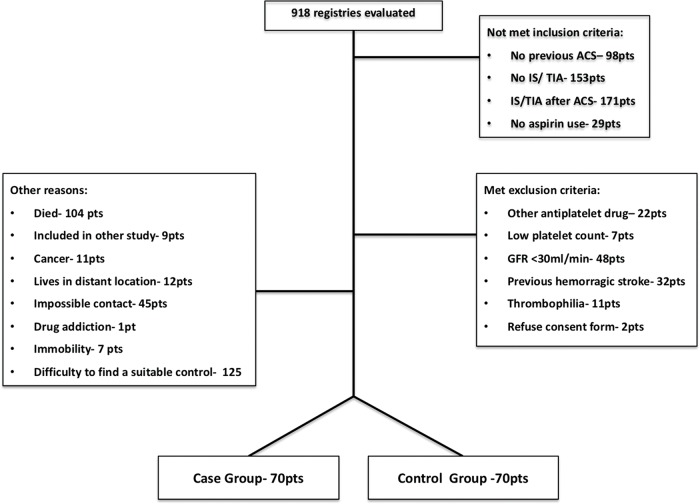
Inclusion Flowchart. ACS- acute coronary syndrome, IS- ischemic stroke, TIA- transient ischemic attack, GFR- glomerular filtration rate, pts- patients.

Participants: The medical records of all patients who were diagnosed with ACS and had a history of IS/TIA, which were included in the coronary care unit and cardiac surgery service databanks, were analyzed. From January 2013 to April 2015, 918 records were evaluated; 412 did not meet the inclusion criteria (mainly because the IS/TIA occurred after the ACS diagnosis), 122 presented at least one of the exclusion criteria (mainly severe kidney dysfunction), and 314 were excluded for other reasons (mainly for the difficulty in finding a suitable matched control subject). As shown in Figure 1, 140 individuals were included in the present study, of whom 70 were in the case group and 70 were in the control group.

Interventions: After the careful review of medical records and confirmation of inclusion and exclusion criteria, eligible patients were invited to participate in the study. After signing the informed consent form, patients underwent a clinical evaluation to confirm the data records and adherence to AAS.

Laboratory tests: In addition to hematological (CBC) and metabolic (renal function, lipid and glucose profile) evaluations, laboratory tests directly related to the main purpose of this study were performed as follows:

- Platelet aggregability - platelet aggregation mediated by thromboxane A2 (TX A2) was assessed by VerifyNow Aspirin®, while platelet aggregation mediated by ADP was evaluated by VerifyNow P2Y12®. The high residual platelet reactivity (HRPR) to AAS was defined by an ARU >550 11.
- Coagulation – fibrinogen and thromboelastography (Reorox®) were evaluated.
- Endogenous fibrinolysis - D dimer and plasminogen activator inhibitor-1 (PAI-1) were evaluated.

### Statistical analysis

Data normality was tested by the Kolmogorov-Smirnov test. Continuous variables are expressed as the mean (+/- SD). Comparisons between the two groups were performed using Student's t-test for Gaussian variables and the Mann-Whitney U test for non-Gaussian variables.

Categorical variables are expressed as the absolute number and relative frequency, and comparisons between the two groups were performed using the Chi-square test or Fisher's exact test, as indicated.

### Multivariable models

In stepwise logistic regression analysis, previous IS/TIA was the dependent variable, and the baseline variables depicted in Table 1 were the independent variables. Each of the platelet, coagulation and endogenous fibrinolysis tests was separately included as an independent variable, thus producing a total of 6 adjusted models.

SPSS version 20.0 software (IBM, USA) was utilized for the analyses, and a *p*-value <0.05 (2-tailed) was used to indicate statistically significant differences. With respect to missing data, we use a listwise deletion approach.

## RESULTS

### Study population

Of the total 140 patients included in the analyses, 70% were male, 68% had a previous AMI, and the index ACS occurred 5 years before inclusion in the study. Furthermore, the mean age of the patients was 60 years.

### Baseline characteristics

As shown in Table 1, patients in the case and control groups were adequately matched for the prespecified variables. However, patients in the case group presented significantly higher systolic blood pressure levels (135.84±16.09 *vs* 123.68±16.11, *p*<0.001), even though the use of antihypertensive medications was more common in this population (2.37±1.09 *vs* 3.0±1.23, *p*=0.006).

Cardiologic risk factors in both groups did not show statistically significant differences. In the analysis of preexisting cardiovascular diseases, there were no significant differences between the two groups with regard to AF, PAD and previous PCI. However, the case group more frequently had a past history of CABG (25.71% *vs* 10%, *p*=0.015).

All patients were taking aspirin, as this was mandatory for participation in the study. Few individuals were using a dosage higher than 100 mg/day. There were no statistically significant differences regarding previous medication usage, except for calcium channel blockers, which were more frequently used in the case group (42.86% *vs* 24.29%, *p*=0.02).

The values for hematological and inflammatory variables were similar between patients in both groups. In relation to the metabolic profile, the lipid profile did not present any differences between the two groups. However, the case group presented higher creatinine levels (but similar values for the glomerular filtration rate) and higher fasting glucose levels (although similar glycosylated hemoglobin levels) compared to the control group.

### Platelet aggregability

Platelet aggregation mediated by thromboxane A2, which was evaluated by VerifyNow® Aspirin, was not statistically significantly different between the groups. Furthermore, this same pattern was observed for the platelet aggregation mediated by ADP, which was evaluated by VerifyNow P2Y12®. Upon analyzing the high residual platelet reactivity to AAS, no significant differences were again observed between the two groups (Table 2).

### Coagulation

The fibrinogen values of the case and control groups were not statistically significantly different, and a similar pattern was observed for the maximum clot firmness, which was evaluated by thromboelastography (Table 2).

### Endogenous fibrinolysis

No statistically significant differences were observed in either the D dimer or PAI-1 levels (Table 2).

### Adjusted models

Table 3 shows the variables that were observed as being significantly associated with a previous IS/TIA in each of the adjusted models. The triglyceride values were associated with a previous IS/TIA in model 1 (including VerifyNow Aspirin), model 2 (including VerifyNow P2Y12) and model 5 (including PAI-1). Hemoglobin values were also significantly associated with a previous IS/TIA in 2 of the models (models 2 and 5). In model 4 (including maximum clot firmness by thromboelastography), systolic blood pressure was associated with a previous IS/TIA. As shown in Table 3, none of the variables in models 3 and 6 (which included fibrinogen and D-dimer, respectively) were associated with a previous IS/TIA. The most important finding was that none of the platelet aggregability, coagulation or fibrinolysis tests were independently associated with a previous cerebrovascular ischemic event.

## DISCUSSION

The main finding of our study is that the higher bleeding risk related to patients with CAD and previous cerebrovascular ischemic events could not be explained by differences in platelet aggregability, coagulation or endogenous fibrinolysis in these patients.

The ADAPT-DES study showed that HRPR to AAS (ARU>550) is a protective factor of major bleeding after drug-eluted stent implantation. In our patient population, HRPR to AAS was similar among patients with previous IS/TIA and those without previous IS/TIA, which does not justify the difference in bleeding risk between these groups of individuals 12.

An exacerbated response to clopidogrel (PRU<95) was associated with higher major bleeding rates in patients included in the ADAPT-DES study. In our study, there were no significant differences observed in the VerifyNow P2Y12® results between patients in the IS/TIA and non-IS/TIA groups who did not utilize clopidogrel. Despite the fact that we did not analyze the eventual antiplatelet response to clopidogrel, the subanalysis of the POPULAR Trial did this, analyzing patients with and without previous IS/TIA, and the results indicated that there were no significant differences in the PRU after clopidogrel use between the groups 13.

It is important to remember that many medications used in ACS treatment influence coagulation factors, which can lead to different bleeding outcomes. Moreover, there seems to be a stronger correlation between serum coagulation factor values and ischemic events than between serum coagulation factor values and bleeding events. At this moment, the only coagulation factor that is clearly associated with a higher incidence of bleeding outcomes is Factor XIII, which was not measured in our study 14.

Although endogenous fibrinolysis is an opposite process to that of atherothrombosis, the correlation between serum values of fibrinolysis markers and ischemic or bleeding events is not clear. In this study, we observed no significant differences in the analyzed groups regarding PAI-1 and D dimer values. This finding suggests that fibrinolysis (at least when measured by the PAI-1 and D dimer values) does not justify the higher bleeding outcomes observed in patients with a previous IS/TIA 15.

Three of the adjusted models used in our study suggested a relationship between low triglyceride levels and a previous IS/TIA. After corroborating our findings with those of the Rotterdam study, a strong correlation between low triglyceride levels and intracerebral hemorrhage was observed 16. In another study, Bonaventura and Cols observed a risk for hemorrhagic stroke in patients with low triglyceride levels (<0.94 mmol/L) that was twice as high as the risk for hemorrhagic stroke in those without low triglyceride levels 17.

There are some possible explanations for these findings. First, some studies have suggested a positive relationship between high triglyceride levels, coagulation factors and blood viscosity, resulting in a possible prohemorrhagic state in patients with low triglyceride values 16,18. Second, cholesterol and fatty acids are essential elements of cell membranes. Experimental studies have suggested a relationship between low cholesterol levels, higher cellular membrane permeability 19 and endothelial weakness in small intracerebral arteries 20. However, the influence of triglyceride levels in this scenario has not been well studied.

Hypertension is an important risk factor for ischemic events, including IS/TIA 21. However, hypertension is correlated with bleeding events, especially during antithrombotic treatment 22. In our study, patients with previous cerebrovascular ischemic events presented higher systolic arterial blood pressure values at baseline despite using more anti-hypertensive medications. These findings suggest a difficulty in controlling systolic hypertension among patients with a previous IS/TIA, which could be related to the higher risk for adverse events in this population.

A low hemoglobin value is a strong predictor of major bleeding events in patients with ACS in the intrahospital phase and long-term follow-up phase 23-26. In our study, low hemoglobin values were independently associated with a previous IS/TIA (individuals at a high risk for bleeding). In addition to being a possible marker for patients suffering from a previous hemorrhage (e.g., gastrointestinal bleedings), hemoglobin is directly associated with primary hemostasis via platelet adhesion, activation and aggregability 27-30.

In summary, the higher risk for bleeding demonstrated among patients with IS/TIA when subjected to more potent anti-thrombotic therapies could not be explained by the variables analyzed in this present study. Therefore, other pathophysiologic mechanisms should be responsible for these findings, as the presence of a dysfunctional hematoencephalic barrier has been demonstrated in individuals with previous cerebrovascular events 31-33.

Our study has some limitations. First, it has a retrospective design. Therefore, despite having case-control matched for 4 different variables, other nonmatched variables could have influenced the results. However, because of the low incidence of a previous IS/TIA in patients with ACS, a prospective design would be unpractical. Second, the case group presented with mild sequelae (mean modified Rankin scale score=2), which may be justified by the worse outcome of patients with higher Rankin scores. Therefore, no conclusion can be drawn for patients with more severe sequelae post-IS/TIA.

## CONCLUSIONS

Platelet aggregability, coagulation and endogenous fibrinolysis showed similar results among CAD patients with and without previous IS/TIA. Therefore, it remains necessary to identify other targets to explain the higher bleeding risk presented by these patients.

## AUTHOR CONTRIBUTIONS

Barbosa CJDG, Nicolau JC and Barreiros RS designed the study. Franci A, Arantes FBB, Furtado RHM, Strunz CMC, Rocha TRF and Baracioli LM acquired data, drafted the manuscript and approved its final version. Ramires JAF, Kalil-Filho R and Nicolau JC provided substantial contributions to the study conception/design, revised the intellectual content of the manuscript and approved its final version.

## Figures and Tables

**Table 1:** Baseline characteristics of the population.

	Case Group	Control Group	95% CI	*p*
Age (years ±SD)	67.64±9.62	66.50±9.97	(-4.42; 2.13)	0.49
Male (%)	71.43	68.57	(-18.03; 12.32)	0.71
White (%)	60	47.14	(-3.53; 29.24)	0.12
BMI (kg/m^2^±SD)	27.86±5.6	27.47±4.46	(-1.33; 2.11)	0.65
HR (bpm±SD)	64.28±8.83	65.91±8.83	(-4.73; 1.46)	0.29
SBP (mmHg±SD)	135.84±16.09	123.68±16.11	(6.19; 18.14)	<0.001
DBP (mmHg±SD)	77.03±10.99	75.66±9.26	(-2.07; 4.80)	0.43
Diabetes (%)	48.57	44.29	(-12.22; 20.79)	0.61
Hypertension (%)	94.29	85.71	(-1.27; 18.41)	0.09
Antihypertensive medications (n±SD)	2.37±1.09	3.0±1.23	(-1,02; -0,24)	0.006
CKD (%)	52.86	37.14	(-0.56; 31.99)	0.61
Hypercholesterolemia (%)	70	80	(-24.25; 4.25)	0.17
Current smoker (%)	5.71	5.71	(-7.69; 7.69)	1.00
Atrial fibrillation (%)	7.14	2.86	(-2.90; 11.47)	0.44
PAD (%)	2.86	5.71	(-9.55; 3.84)	0.68
AMI (%)	67.15	70	(-18.23; 12.52)	0.51
Time since ACS (years±SD)	5.57±2.78	5.05±2.67	(-0.40; 1.43)	0.27
PCI (%)	31.43	31.43	(-15.38; 15.38)	1.00
CABG (%)	25.71	10	(3.30; 28.13)	0.015
**Medications**				
Aspirin >100 mg/d (%)	11.42	8.57	(-7.07; 12.78)	0.57
Statin (%)	94.29	94.29	(-7.69; 7.69)	1.00
ACE inhibitor (%)	54.29	57.14	(-19.31; 13.59)	0.73
ARB (%)	27.14	18.57	(-5.27; 22.41)	0.22
Calcium channel blocker (%)	42.86	24.29	(3.23; 33.91)	0.02
Diuretics (%)	44.29	37.14	(-9.09; 23.38)	0.38
Nitrate (%)	28.57	15.71	(-0.73; 26.45)	0.06
Beta blocker (%)	88.57	80.00	(-3.40; 20.54)	0.16
Oral hypoglycemic medications (%)	41.43	44.29	(-19.25; 13.53)	0.73
Insulin (%)	10.00	18.57	(-20.08; 2.93)	0.14
PPI (%)	61.43	42.86	(2.31; 34.83)	0.07
Oral anticoagulants (%)	7.14	2.86	(-2.90; 1.47)	0.44
SRI (%)	12.86	7.14	(-4.18; 15.61)	0.25
**Hematologic, metabolic and inflammatory tests**				
Hemoglobin (g/dL)	14.18±1.63	14.59±1.40	(-0.93; 0.09)	0.10
Platelets/mm^3^	242228.6±62225.98	237857.1±56653.1	(-16411; 25154)	0.67
Leukocytes/mm^3^	7468.29±1882.81	7647.57±1961.47	(-821.80; 463.28)	0.58
Total cholesterol (mg/dL)	162.56±38.86	165.61±38.44	(-15.98; 9.86)	0.64
LDL (mg/dL)	92.39±30.01	93.23±31.51	(-11.13; 9.44)	0.87
HDL (mg/dL)	43.37±13.2	44.31±9.93	(-4.85; 2.96)	0.63
Triglycerides (mg/dL)	133.00±59.57	144.46±95.69	(-38.10; 15.18)	0.90
BUN (mg/dL)	44.26±14.03	41.46±15.17	(-2.08; 7.68)	0.09
Creatinine (mg/dL)	1.24±0.35	1.11±0.27	(0.02; 0.23)	0.037
GFR (mL/min/1.73 m^2^)	66.03±22.61	68.48±21.54	(-9.88; 4.98)	0.35
Fasting glucose (mg/dL)	116.16±32.03	134.88±57.58	(-62.49; -2.32)	0.031
HBA1c (%)	7.57±8.74	7.05±2.17	(-1.61; 2.65)	0.63
Lipoprotein (a)	47.39±43.81	44.14±44.78	(-11.73; 18.23)	0.55
us-CRP (mg/dL)	5.41±7.95	3.79±5.25	(-0.63; 3.88)	0.06
INR	1.07±0.21	1.08±0.31	(-0.098; 0.078)	0.36

BMI- body mass index, HR- heart rate, SBP- systolic blood pressure, DBP- diastolic blood pressure, CKD- chronic kidney disease, PAD- peripheral artery disease, AMI- acute myocardial infarction, PCI- percutaneous coronary intervention, CABG- coronary artery bypass graft, ACE- angiotensin-converting enzyme, ARB- angiotensin receptor blocker, PPI- proton-pump inhibitors, SRI- serotonin receptor inhibitors, LDL- low-density lipoprotein cholesterol, HDL- high-density lipoprotein cholesterol, BUN- blood urea nitrogen, GFR- glomerular filtration rate, HBA1c- glycated hemoglobin, us-CRP- ultra sensitive C-reactive protein, INR- international normalized ratio, 95% CI- 95% confidence interval.

**Table 2:** Platelet aggregation, coagulation and endogenous fibrinolysis.

	Case Group	Control Group	95% CI	*p*
**Platelet aggregation mediated by thromboxane A2**				
VerifyNow Aspirin^^®^^ (ARU±SD)	525.00±79.78	530.35±83.81	(-32.9; 22.2)	0.7
HRPR to AAS (%)	40.58	42.03	(-0.15; 0.17)	0.86
**Platelet aggregation mediated by ADP**				
VerifyNow P2Y12^^®^^ (PRU±SD)	262.14±43.03	251.74±43.72	(-6.16; 26.97)	0.21
**Coagulation**				
Fibrinogen (mg/dL±SD)	370.46±80.11	366. 86±94.19	(-25.62; 32.82)	0.8
MCF (Pa±SD)	2136.00±569.97	2001.27±635.68	(-67.87; 337.33)	0.19
**Endogenous fibrinolysis**				
D dimer (ng/dL±SD)	645.29±1321.84	459.24±769.54	(-175.40; 547.52)	0.72
PAI-1 (pg/mL±SD)	31.78±30.53	27.42±20.13	(-4.40; 13.13)	0.73

ARU- aspirin reaction units, HRPR- high residual platelet reactivity, ADP- adenosine diphosphate, PRU- P2Y12 reaction units, MCF- maximum clot firmness, PAI-1- plasminogen activator inhibitor, SD- standard deviation, 95% CI- 95% confidence interval.

**Table 3:** Variables that correlated significantly and independently with previous IS/AIT in each of the adjusted statistical models.

Adjusted Model	Variables	*p*	OR	95% CI
Model 1				
	Triglycerides	0.02	0.98	0.97-0.99
Model 2				
	Triglycerides	0.01	0.98	0.96-0.99
	Hemoglobin	0.04	0.35	0.12-0.95
Model 3				
	N.V	N.V	N.V	N.V
Model 4				
	SBP	0.03	2.69	1.05-6.8
	DBP	0.04	0.36	0.13-0.98
Model 5				
	Triglycerides	0.03	0.98	0.97-0.99
	Hemoglobin	0.03	0.11	0.01-0.87
Model 6				
	N.V	N.V	N.V	N.V

Model 1- includes VerifyNow Aspirin^^®^^, Model 2- includes VerifyNow P2Y12^^®^^, Model 3- includes fibrinogen, Model 4- includes the maximum clot firmness by thromboelastogram, Model 5- includes PAI-1, Model 6- includes D-dimer, N.V- no statistically significant variables in the adjusted model, SBP- systolic blood pressure, DBP- diastolic blood pressure, 95% CI- 95% confidence interval.
